# Predicting protein function via downward random walks on a gene ontology

**DOI:** 10.1186/s12859-015-0713-y

**Published:** 2015-08-27

**Authors:** Guoxian Yu, Hailong Zhu, Carlotta Domeniconi, Jiming Liu

**Affiliations:** 1 College of Computer and Information Sciences, Southwest University, Beibei, Chongqing, China; 2 Key Laboratory of Symbolic Computation and Knowledge Engineering of Ministry of Education, Jilin University, Changchun, China; 3 Department of Computer Science, Hong Kong Baptist University, Hong Kong, Hong Kong; 4 Department of Computer Science, George Mason University, Fairfax, VA USA

**Keywords:** Function prediction, Downward random walk, Gene ontology, Partially annotated proteins

## Abstract

**Background:**

High-throughput bio-techniques accumulate ever-increasing amount of genomic and proteomic data. These data are far from being functionally characterized, despite the advances in gene (or gene’s product proteins) functional annotations. Due to experimental techniques and to the research bias in biology, the regularly updated functional annotation databases, i.e., the Gene Ontology (GO), are far from being complete. Given the importance of protein functions for biological studies and drug design, proteins should be more comprehensively and precisely annotated.

**Results:**

We proposed *d*ownward *R*andom *W*alks (dRW) to predict missing (or new) functions of partially annotated proteins. Particularly, we apply downward random walks with restart on the GO directed acyclic graph, along with the available functions of a protein, to estimate the probability of missing functions. To further boost the prediction accuracy, we extend dRW to dRW-*k*NN. dRW-*k*NN computes the semantic similarity between proteins based on the functional annotations of proteins; it then predicts functions based on the functions estimated by dRW, together with the functions associated with the *k* nearest proteins. Our proposed models can predict two kinds of missing functions: (i) the ones that are missing for a protein but associated with other proteins of interest; (ii) the ones that are not available for any protein of interest, but exist in the GO hierarchy. Experimental results on the proteins of Yeast and Human show that dRW and dRW-*k*NN can replenish functions more accurately than other related approaches, especially for sparse functions associated with no more than 10 proteins.

**Conclusion:**

The empirical study shows that the semantic similarity between GO terms and the ontology hierarchy play important roles in predicting protein function. The proposed dRW and dRW-*k*NN can serve as tools for replenishing functions of partially annotated proteins.

**Electronic supplementary material:**

The online version of this article (doi:10.1186/s12859-015-0713-y) contains supplementary material, which is available to authorized users.

## Background

The Gene Ontology (GO) is a controlled vocabulary of terms for describing the biological roles of genes and their products (i.e., proteins) [1]. GO organizes the ontological knowledge (or GO terms) in a direct acyclic graph (DAG), and represents the knowledge in three orthogonal sub-ontologies, namely a Biological Process (BP), a Molecular Function (MF) and a Cellular Component (CC). In the DAG, a GO term describes a more specific functional role than its ancestor terms. If a protein is annotated with a specific term, then it is also annotated with the corresponding ancestor terms. This rule is known as the *true path rule* [1, 2]. Hereinafter, we use the word “function” as synonymous of ‘GO term’ or ‘term’, regardless of the sub-ontology the term belongs to.

The advance in protein functional annotation far lags behind the pace of accumulated proteomic and genomic data. The Human Proteome Project consortium recently claimed that we still have very little information about the cellular functions of approximately two-thirds of human proteins [3]. Schones et al. [4] found that the functional annotations of high-throughput genomic and proteomic data are biased and shallow. Therefore, automatically annotating the functional roles of these proteins using GO terms can facilitate the understanding of life at the cellular level, and lay a foundation for the development of diagnostic, prognostic, therapeutic, and preventive medical applications [3, 5, 6].

Although researchers have been working on protein function prediction for more than ten years, the functional roles of proteins are still poorly characterized for several reasons: (i) most functional information is yet to be discovered through direct experiments in the first place [7], and it is time consuming and costly to annotate proteins in wet-labs; (ii) the wet-lab verified functions of proteins are still limited by the experimental techniques and the biological research interest [4, 8–10]. These techniques often can only provide partial annotations, which are not specific enough to be of biological interest [4] and result in the issues of evaluation of predicted functions [11, 12]; (iii) our current knowledge of the GO terms and structure is incomplete. Both are updated regularly; as a result, some GO terms are obsolete and some new ones are included from time to time. For these reasons, developing computational models to comprehensively annotate proteins is of great importance and necessity. More importantly, these models should explicitly take into account the incomplete functional annotation of proteins.

Various computational models have been proposed for protein function prediction and their feasibility has been shown [5, 6, 13]. Most of these methods [2, 14–18] explicitly (or implicitly) assume that the available functional annotations of proteins are complete, and make use of the annotations to predict the functions of unlabeled proteins. Thus, these methods ignore the fact that proteins are partially annotated and they cannot replenish (or predict) functions of a partially annotated protein. Given that the current functional annotation of proteins are shallow and far from being complete [4, 5], and given the true path rule, it is more desirable to know the specific functions of a protein, rather than the general ones. For example, ‘GO:0072576’ (liver morphogenesis) is a descendant of ‘GO:0001889’ (liver development); thus, a protein annotated with ‘GO:0072576’ provides more biological information than this protein annotated with ‘GO:0001889’. In this paper, we investigate the possibility of replenishing missing but more informative functions of a protein from the currently annotated functions of the same protein.

Predicting functions of proteins that already have some functional annotations has been set as a new challenge in the second large-scale community-based critical assessment of protein function annotation (CAFA) [5, 19]. A few approaches explicitly consider the incomplete annotations of proteins, and predict missing annotations for partially annotated proteins, or for completely unlabeled proteins [20–23]. Yu et al. [20] used co-expression data and an edge-based functional terms’ taxonomy similarity to determine specific functions of a protein. At first, all functions associated with the *k* nearest neighborhood proteins are chosen as candidate functions of a protein. These candidate functions are weighted based on their taxonomy similarity and their frequency. Eventually, the resulting representative functions are assigned to the protein. Zhu et al. [21] extended the method proposed by Yu et al. [20] via integrating gene co-expression data with PPI networks to filter the interacting proteins of a target protein, and to enhance the degree of function consensus among the neighbors of a protein. Similarly, they used the functional annotations of the filtered neighborhoods and an edge-based taxonomy similarity to predict the functions of the target protein. King et al. [24] directly used the annotation patterns of proteins to train a decision tree classifier and a Bayes classifier for function prediction. These two classifiers need sufficient annotations for training, and they do not work well for *sparse* GO terms, which are associated with very few (≤10) proteins. Such GO terms constitute the majority. To avoid this limitation, Tao et al. [22] introduced an approach called information theory semantic similarity (ITSS). ITSS first measures the semantic similarity between pairwise GO terms based on a taxonomy, which is similar to Lin’s similarity [25]. Based on the semantic similarity between two GO terms, ITSS computes the semantic similarity between two proteins by averaging the pairwise similarities between the reciprocal GO terms associated with the two respective proteins. ITSS then employs a simple *k*NN classifier based on the semantic similarity between proteins to predict the functions. Dong et al. [26] utilized the vector space model and latent semantic indexing on a protein-function association matrix for function prediction. These methods can only assign functions that are associated with the neighbors (or some other proteins) to a protein.

**Fig. 1:**
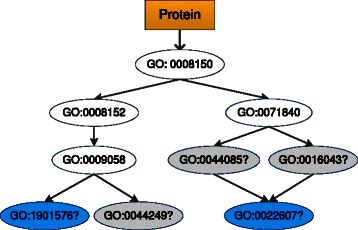
An example of a partially annotated protein. The GO terms in the white ellipses are the currently available functions of the protein, and the terms in the colored ellipses are the missing functions of the protein. In particular, the terms in the grey ellipses are missing functions of the *first* type: they are associated with other proteins, but are missing for the protein being considered. The terms in the blue ellipses belong to the *second* type: they exist in the GO hierarchy, but they are not associated with any protein of interest. We observe that any missing function of a protein should be a leaf node of the hierarchy, and this hierarchy is defined with respect to the available terms associated with the protein, rather than with the whole GO hierarchy. We can replenish a non-leaf term of a protein directly using its descendant terms, due to the true path rule of GO

A protein often engages in several cellular processes and thus is annotated with several GO terms. Each term can be viewed as a functional label, and protein function prediction can be modeled as a multi-label learning problem [27–32]. From this viewpoint, protein function prediction using partial annotations can be modeled as a multi-label and weak-label learning problem [23, 33]. In multi-label weak-label learning, a multi-label instance is partially labeled, and some of its ground-truth labels are not available (or missing). The goal of weak-label learning is to replenish the missing labels and to predict the labels of new instances using the partially labeled ones. Yu et al. [23] proposed a weak-label learning method called protein function prediction using weak-label learning (ProWL). ProWL uses the Cosine similarity to measure the correlation between two functional labels. The available functional annotations of a protein, along with the correlation between functions, are used to estimate the likelihood of missing functions. In addition, ProWL uses a network-based classifier to exploit PPI networks to replenish missing functions of a partially annotated protein, and to predict functions for an unlabeled protein. Yu et al. [34] assumed that the functional annotation of a protein depends on its feature information, and suggested a weak-label learning based method called ProDM. ProDM maximizes the dependency between the features and the functional annotations of proteins to make prediction. These weak-label learning methods explicitly consider only the flat relationships among labels. More recently, Yu et al. [35] proposed a method called PILL to predict protein functions using incomplete hierarchical labels. PILL takes advantage of the hierarchical and flat relationships among functional labels, along with the PPI network to replenish the missing annotations of partially annotated proteins. PILL significantly outperforms the aforementioned weak-label learning methods, and shows the paramount importance of using hierarchical relationships among functional labels. Sefer and Kingsford [18] suggested an Metric Labeling method to optimize the distance between functions by using the GO structure information, and then to predict function for completely unlabeled proteins. However, none of the aforementioned methods can assign a GO term, which exists in the GO hierarchy but has not yet been associated with any protein of interest (i.e., the GO terms in the blue ellipses of Fig. 1), to a protein.

In this paper, we propose *d*ownward *R*andom *W*alks (dRW) and its variant dRW-*k*NN to replenish functions of a partially annotated protein. dRW and dRW-*k*NN can predict two kinds of missing functions: (i) Functions that should be associated with a protein, but they are currently missing, e.g., the GO terms in the grey ellipses in Fig. 1; (ii) Functions that exist in the GO hierarchy and should be associated with some proteins, but they are currently not associated with any, i.e., the GO terms in the blue ellipses in Fig. 1. Some pioneers apply text mining techniques (i.e., GOAnnotator [36]) on the biomedical texts to predict the second kind of functions [37]. Nevertheless, it is important to highlight that the prediction of the second kind of functions, to the best of our knowledge, is still less studied in computational model-based protein function prediction.

## Methods

Let *N* be the number of proteins and $|\mathcal {T}|$ the number of GO terms. Lets assume the *i*-th protein is annotated with the terms in $\mathcal {T}_{i}$ ($\mathcal {T}_{i} \subset \mathcal {T}$). Furthermore, there exist terms in $\mathcal {T}$ that are not associated with any proteins. Our goal is to determine whether protein *i* should be annotated with a term $t \in \mathcal {T}$, where $t\not \in \mathcal {T}_{i}$. To achieve this goal, we introduce dRW on the GO DAG to pre-estimate the likelihood that the protein should be annotated with *t*. A random walk on a graph is often described by a transitional probability matrix. We define the transitional probability between nodes (each node corresponds to a GO term) in the DAG based on their semantic similarity. Various semantic similarity measures have been proposed to compute the similarity between two GO terms [8, 9, 11, 25, 38–41]. These similarity measures focus on different characteristics of the GO structure and compute the similarity between two terms or two groups of terms. A comprehensive coverage of these semantic similarities is out of scope in this paper. For more information on semantic similarities, the reader can refer to [8, 9, 39–41] and references therein.

Here we first introduce a structure-based semantic similarity to measure the similarity between two GO terms. Second, we introduce dRW on the GO DAG to estimate the likelihood that a term is missing for a protein. Next, we measure the semantic similarity between two proteins based on their GO annotations and the structure-based similarity. Then, dRW-*k*NN is introduced to replenish functions of a protein based on the functions pre-estimated by dRW and the functions associated with its neighborhood proteins.

### Structure based semantic similarity

We take advantage of a structure-based semantic similarity, which is a variant of Lin’s similarity [25] to measure the similarity between two GO terms. Lin’s similarity is defined as follows:

(1) $$ sim\left(t_{1},t_{2}\right)= \frac{2 \times IC\left(t^{\ast}\right)}{IC\left(t_{1}\right)+IC\left(t_{2}\right)}   $$

where *t* ^∗^ is the most informative common ancestor term of *t*_1_ and *t*_2_, *t* ^∗^ subsumes both *t*_1_ and *t*_2_, if *t*_1_ is the ancestor of *t*_2_, then *t* ^∗^ is *t*_1_. *I* *C*(*t* ^∗^) is the structure-based information content (IC) of term *t* ^∗^. There are alternative ways to define the similarity between *t*_1_ and *t*_2_, we choose Lin’s similarity for its wide application and empirical good performance in the previous study [15, 18, 35].

To reduce the bias of incomplete annotations and to produce consistent information content of the terms across different species, similarly to Tao et al. [22] and Teng et al. [40], we compute *I* *C*(*t*) of a GO term *t* using the number of its descendants in the GO hierarchy, rather than the frequency of this term. *I* *C*(*t*) is inversely proportional to the number of descendants of *t*, because the more descendants *t* has, the less specific it is. *I* *C*(*t*) is defined as:

(2) $$\begin{array}{@{}rcl@{}} IC(t)&=&\frac{log\left((1+|desc(t)|)/|\mathcal{T}|\right)}{log(1/|\mathcal{T}|)}\\ &=&(1-\frac{log(1+ |desc(t)|)}{log |\mathcal{T}|})  \end{array} $$

where *d* *e* *s* *c*(*t*) is the set of descendant GO terms of *t* and |*d* *e* *s* *c*(*t*)| is the cardinality of *d* *e* *s* *c*(*t*). If Eq. (2) use the frequency of term *t* to define *I* *C*(*t*), then for a GO term that is not associated with any of the *N* proteins, its Lin’s similarity with respect to other GO terms is set to 0. However, the similarity between *t* and other terms should not be set as 0.

Hereinafter, Lin’s similarity with *I* *C*(*t*) defined by Eq. (2) is called as Lin’s structure similarity, and Lin’s similarity with *I* *C*(*t*) defined by the frequency of *t* is named as Lin’s corpus similarity. Our choice of Lin’s structure similarity is driven by the fact that functional annotations of proteins are incomplete and biased to the biologist research interest [4, 8, 12], the frequency of a term is often coarsely estimated. Although Eq. (2) equally treats all the GO terms, we observe that dRW based on Lin’s structure similarity achieves better performance than Lin’s corpus similarity, and it also gets better results than dRW based on a recently proposed semantic similarity that takes advantage of disjointness between terms to define the similarity between GO terms [41].

### Estimatingmissing functions using downward random 274 walks (dRW)

We introduce downward random walks with restart [42] on the GO DAG to estimate missing functions of proteins. There are several rationalities to apply downward random walks on the DAG. (i) From the *true path rule* of GO annotations [1, 2], if a protein is annotated with a specific GO term, then it is also annotated with the corresponding ancestor terms, and may or may not be annotated with the descendant terms. (ii) The missing functions of a protein are the descendants of the terms associated with the protein. For example, ALG6 was annotated with 43 BP GO terms by 2010-01-20 and it was annotated with 47 terms by 2014-06-09. The 4 missing functions of ALG6 are the descendants of GO:0006464 and GO:0006487, which were associated with ALG6 by 2010-01-20. CLDN16 was associated with 24 BP GO terms by 2010-01-20 and it was annotated with 34 terms by 2014-06-09. The 10 missing functions of CLDN16 are the descendants of GO: 0008150 and GO: 0044699, which were associated with CLDN16 by 2010-01-20. The functional annotations of these two proteins are illustrated in Fig. S1 of Additional file 1. (iii) Schones et al. [4] observed that the functional annotations of proteins from high-throughput experiments are often shallow, and these proteins should be annotated with more specific functions. Given that, a downward random walker that starts from the available terms associated with a protein has the potential of identifying additional functions of the same protein.

Let $A \in \mathbb {R}^{|\mathcal {T}| \times |\mathcal {T}|}$ be the association matrix of the DAG graph. If *t*_2_ is a child of *t*_1_, *A*(*t*_1_,*t*_2_)=1, otherwise *A*(*t*_1_,*t*_2_)=0. To stimulate the random walk on the DAG, we need to filter out transitions between pairs of GO terms that do not have a parent-child relationship. We use *f* *s* *i* *m*(*t*_1_,*t*_2_)=*s* *i* *m*(*t*_1_,*t*_2_)×*A*(*t*_1_,*t*_2_) to represent the filtered semantic similarity between *t*_1_ and *t*_2_. Obviously, if *A*(*t*_1_,*t*_2_)=0, i.e., *t*_1_ is not the parent of *t*_2_, then there is no transition between the two.

This filter process is based on the observation that the conditional probability that a protein is annotated with *t*, given that the protein is already annotated with *t*’s parent terms, is much larger than the conditional probability that the protein is annotated with *t*, given that it is annotated with *t*’s other ancestor terms [35]. Based on *fsim*, we can define the normalized *initial* transition probability between two GO terms as

(3) $$ W\left(t_{1}, t_{2}\right)=\frac{fsim\left(t_{1}, t_{2}\right)}{\sum_{t \in \mathcal{T}} fsim\left(t, t_{2}\right)}.   $$

Suppose a random walker starts from a GO term *t*. The walker iteratively reaches its descendant *v* (if any) according to the corresponding transition probabilities. At the same time, the walker also has some probability to stay at *t*. Let *R*_*s*_(*t*,*v*) be the probability the walker starts at *t* and then stays at *v* during the time step *s*. Then *R*_0_(*t*,*t*)=1 and *R*_0_(*t*,*v*)=0 for each *t*≠*v*. *R*_*s*+1_(*t*,*v*) is computed as:

(4) $$ R_{s+1}(t,v)= \eta \sum_{u\in \left(t \cup desc(t)\right)} R_{s}(t, u) W(u, v) + (1-\eta) \mathbf{e}_{t}   $$

where **e**_*t*_ is a $|\mathcal {T}| \times 1$ start vector with the *t*-th element set to 1, and all others set to 0; *η*∈[0,1] is the restart probability, and 1−*η* is the probability for the walker to stay at *t*. The walker performs direct random walks with restart on the GO DAG with limited number of direct edges, since the maximum depth of GO terms in the whole GO hierarchy (as of July 2014) is 15, *t* will reach a steady state after several iterations. In our empirical study, we set the number of iterations to 10.

A walker starting from a GO node *t* can move to its descendant GO nodes (if any) and can also stay at *t*. As the walker walks along the GO DAG, since the transition probability between two terms is filtered by the GO structure and the probability for a walker stays at the starting GO node is not zero, the probability the walker starts from *t* to its direct child GO nodes (if any) is larger than that to *t*’s other descendant nodes (if any). Similarly, the walker will not end in one leaf of the ontology, and the probability a walker moves to a leaf node is smaller than that to the leaf node’s ancestors. If the probability for a walker stays at the starting point is 0 (namely *η*=0 in Eq. (4)), then the random walker will end at one leaf node. For simplicity and avoiding bias, we just set *η*=0.5, which means a random walker having equal probability to stay at the starting GO node and move to the descendant GO nodes of the starting node.

To this end, we make use of the steady transition probability $\tilde {R}$ and available functions of a protein to predict potential missing functions of a protein. Let $\mathcal {T}_{i}$ be the currently available (maybe incomplete) GO terms associated with the *i*-th protein, for a term $v \not \in \mathcal {T}_{i}$, $\tilde {R}(t,v)$ is the stable transition probability from *t* to *v*, and it can be viewed as the estimated likelihood that the *i*-th protein is annotated with *v* also. Taking into account all the available terms in $\mathcal {T}_{i}$, the overall likelihood is:

(5) $$ \mathcal{L}(i, v)=\sum_{t \in \mathcal{T}_{i}} \tilde{R}(t, v) \quad s.t. \ \tilde{R}(t,v)>\theta   $$

where *θ* is an adaptive threshold parameter and is equal to the mean value of all nonzero elements in $\tilde {R}(t,\cdot)({t \in \mathcal {T}_{i}})$. *θ* is adopted to filter out estimations that are too small, since the number of candidate GO terms is rather large and only a few are associated with a given protein. In other words, *θ* can help removing some false positive estimations, since the missing GO terms of a protein are often located at deeper levels of the GO hierarchy, and the closest ancestors (i.e., parent GO terms) can provide more accurate estimations than the farthest ancestors [35]. $\mathcal {L}(i, v)$ in Eq. (5) gives the dRW estimated likelihood of a new function *v* for the *i*-th protein.

Suppose GO term *v* currently is not associated with any of the *N* proteins and it should be associated with the *i*-th protein, if its ancestor terms (i.e., *t*) are associated with the *i*-th protein, since *R*(*t*,*v*)>0, then $\mathcal {L}(i,v)>0$. In this way, dRW can predict the second kind of missing functions, namely the functions that are not associated with any protein of interest. Lin’s structure similarity in Eq. (2) equally treats each GO term, and thus each subbranch of GO maybe equally well refined. However, the available GO annotations of a protein and the GO hierarchy can force the replenished functions not evenly distributed in the subbranch.

### Predicting missing functions using dRW-kNN

The semantic similarity between proteins derived from functional annotations of proteins is correlated with the similarity derived from different types of proteomic data and genomic data [8, 9, 40, 43], for example, gene sequences, co-expressions and PPIs. Some approaches directly utilize the pattern of GO annotations of proteins to predict protein functions [24], and some methods assume that the semantic similarity between proteins can be used to predict additional functions of a partially annotated protein [22, 26].

We use the GO annotations of proteins and Lin’s structure similarity to compute the semantic similarity between two proteins. We then use this semantic similarity to determine the neighborhood relationship among proteins and to predict missing functional annotations of partially annotated proteins. Similarly to Tao et al. [22], the semantic similarity between two proteins *i* and *j* is defined as follows:

(6) $$ psim(i,j)=\frac{2 \times \sum_{\left(t_{1},t_{2}\right)\in \mathcal{P}} sim\left(t_{1},t_{2}\right)}{|\mathcal{T}_{i}|+|\mathcal{T}_{j}|}   $$

where $\mathcal {P}$ represents the set of reciprocal pairs of GO terms between proteins *i* and *j*. Each pair in $\mathcal {P}$ consists of a term from $\mathcal {T}_{i}$ and a term from $\mathcal {T}_{j}$ that are mutually most similar to one another. As such, if $\mathcal {T}_{i}$ and $\mathcal {T}_{j}$ are not empty, $\mathcal {P}$ will also be not empty.

We apply a *k*NN style classifier to predict functions for a partially annotated protein. In particular, we make use of the missing functions pre-estimated by dRW and the functions of the nearest neighbors. The probability that a protein *i* is annotated with term *v* ($v \not \in \mathcal {T}_{i}$) is:

(7) $$ \tilde{\mathcal{L}}(i,v)= \frac{\sum_{j \in \mathcal{N}_{k}(i)} psim(i,j) \times \mathcal{L}(j,v)}{k}   $$

where $\mathcal {N}_{k}(i)$ consists of the *k* nearest proteins to protein *i*, and the neighborhood relationship is determined by *psim* in Eq. (6). Note, $\mathcal {N}_{k}(i)$ always contains the *i*-th protein itself. From Eq. (7), if the *i*-th protein is missing a term, and its neighbors are annotated with such term, then the term can be assigned to the *i*-th protein. Furthermore, if a term *t* is not associated with any of the *N* proteins, but it is available in the GO hierarchy and semantically close to other terms that are associated with some proteins, then *t* can still be replenished. We call the downward random walks approach combined with the *k*NN classifier in Eq. (7) as dRW-*k*NN. Obviously, dRW can be viewed as a special case of dRW-*k*NN when *k*=1.

The proposed dRW and dRW-*k*NN solely depend on the available functional annotations of proteins, they do not take other protein-specific information (i.e., amino acid sequences and protein-protein interactions) as input. If two entirely different proteins with the same initial functional annotations, they will be predicted with the same functions by dRW and dRW-*k*NN. In fact, the proposed dRW-*k*NN can incorporate other kinds of proteomic data (i.e., amino acid sequences and protein-protein interactions) to determine the neighborhood proteins of a protein. In this way, two entirely different proteins with the same initial functional annotations can have different neighborhood proteins and be predicted with different functions. To focus on the main idea of random walks on the GO hierarchy and to keep consistency with other related methods, we do not include other kinds of proteomic data in the experiments. It is an interesting future work to integrate the protein-specific information with dRW-*k*NN and to study the difference between using semantic similarity and protein-specific information.

The Naive method suggested by Clark and Radivojac [30] is a baseline approach in CAFA, it also directly uses the available GO annotations of proteins to predict protein function, and it is quite different from the proposed dRW and dRW-*k*NN. Naive is solely based on the frequency of a function, and it can not replenish the second kind of missing functions. In contrast, dRW and dRW-*k*NN depend on the functional annotations of proteins and the GO hierarchy, and they can predict the second kind of missing functions, and they are not so dependent on the frequency of function as Naive. In addition, dRW-*k*NN utilizes the semantic similarity between proteins to determine the functional annotations of proteins, and the semantic similarity is correlated with different types of proteomic data, thus dRW-*k*NN has the potential to incorporate other proteomic data, whereas Naive does not. Our following experiments will show that dRW and dRW-*k*NN produce quite different results from Naive in the same experiments.

## Results and discussion

### Datasets and experimental setup

We downloaded the GO file^1^ that contains hierarchical relationships between GO terms organized in three orthogonal axes of biological concepts. The Gene Ontology Annotation (GOA) files of Yeast and Human were obtained from the European Bioinformatics Institute^2^. Each GOA file contains annotations relating the genes to the three sub-ontologies, namely BP, MF, and CC. We processed the GO file to exclude the GO terms annotated as ‘obsolete’. We processed the GOA file to exclude the annotations with evidence code ‘IEA’ (Inferred from Electronic Annotation), ‘NR’ (Not Recorded), ‘ND’ (No biological Data available), or ‘IC’ (Inferred by Curator).

The missing functions of a protein are often located at deep levels of the GO hierarchy. These functions are typically associated with no more than 10 proteins and thus are viewed as sparse functions (or GO terms). Many methods are shown to work well for the terms associated with 10 (or more) proteins [2, 15, 23]. Tao et al. [22] reported that no prediction method is able to achieve highly accurate results for sparse terms, and proposed ITSS to predict missing gene functions using sparse terms. We follow a similar preprocess step as ITSS to conduct experiments on sparse terms associated with at least 3 proteins. The GOA file provides the most detailed level GO terms in the ontology that correctly describes the biology of the gene and its products^3^. We apply the true path rule to append all the ancestor GO terms of the detailed terms for a protein. The summary of processed GO annotations of proteins are listed in Table 1. From the Table, we can observe that the sparse terms occupy the largest portion of $\mathcal {T}$, and few terms are associated with more than 30 proteins. An interesting observation is that the average number of terms associated with a protein is close to the standard deviation; this is because some proteins in the GOA are not annotated with any term (excluding the annotations with evidence code ‘IEA’, ‘NR’, ‘ND’, and ‘IC’). For example, 1191 proteins in Yeast and 7094 proteins in Human are not annotated with any BP terms. Another reason is that some proteins are annotated with detailed terms and some others are not. These observations drive us to determine the potential missing functions of these proteins.

**Table 1:** Statistics of GO annotations. The data in parentheses along with Yeast (or Human) is the number of proteins in that dataset. First column: $|\mathcal {T}|$ is the total number of distinct GO terms used for empirical study, and the data in parentheses is the number of GO annotations of all the proteins. [3,10) characterizes the number of terms associated with at least 3 and less than 10 proteins; [10,30) represents the number of terms associated with at least 10 and less than 30 proteins; and ≥30 includes the terms associated with at least 30 proteins, Avg ±Std is the average number of annotations of a protein and its standard deviation. The root GO term in each sub-ontology (BP, CC and MF) are not included

		$|\mathcal {T}| $	[3,10)	[10,30)	≥30	Avg ± Std
Yeast(5914)	BP	2979 (210949)	1350	761	868	35.67 ± 34.62
	CC	731 (79378)	359	170	202	13.42 ± 12.01
	MF	978 (35033)	546	236	196	5.92 ± 6.47
Human(19009)	BP	7294 (694455)	3237	1877	2180	36.53 ± 53.25
	CC	978 (230826)	414	224	340	12.14 ± 12.66
	MF	1772 (106410)	943	420	409	5.59 ± 7.99

There are no off-the-shelf datasets that can be directly used to check the performance of missing functions prediction, since the GO and GOA files are updated regularly. Similarly to the experimental protocol in Yu et al. [35], we assume that the currently available GO annotations of a protein are complete. We iteratively and randomly mask some leaf GO terms of a protein. These masked terms are considered as the missing (or new) functions of the protein, and used to investigate the performance of the proposed models. In the masking process, a non-leaf GO term of a protein can turn to be a leaf term once all its child terms are masked. Some sparse terms may be *completely* masked in the experiments, thus they end up not being associated with any of the *N* proteins, though they exist in the GO hierarchy. These completely masked terms are viewed as the second kind of missing functions.

In the experiments we use *m* to denote the number of missing functions of a protein, *N*_*m*_ to represent the number of masked functions, and $|\mathcal {T}_{m}^{0}|$ to represent the number of the second kind of missing functions of the *N* proteins for a given setting of *m*. For example, *m*=3 means three functions are masked for a protein, *N*_3_=1000 means 1000 functions are masked for the *N* proteins, and $|\mathcal {T}_{3}^{0}|=50$ means 50 functions in $\mathcal {T}$ are not associated with any of the *N* proteins. For the protein that is annotated with no more than *m* functions, we do not mask all the functions, and ensure it has one GO term (if any). A portion of the proteins has no function annotations, and we do not apply the mask operation on these proteins.

### Methods and evaluation metrics

We compare our proposed dRW and dRW-*k*NN against PILL [35], ITSS [22] and Naive [5]. PILL and ITSS were introduced in the section of Background. They can predict the first kind of missing functions of a protein, but they can not make predictions for the second kind (which do not have any associated proteins). Naive is a baseline approach in CAFA, it predicts functions of a protein based on the frequency of functions: the larger the frequency of a function is, the larger the likelihood is for a protein to be annotated with such function. Naive outperforms many competitive function prediction methods [5]. The parameter setting for these methods are provided in Additional file 1.

The accuracy of protein function prediction can be assessed by different evaluation criteria or metrics, and different prediction models are affected by different metrics. To do a fair and comprehensive comparison, we use six evaluation metrics, namely *MacroF1*, *AvgROC*, *RankingLoss*, *RAccuracy*, *Fmax*, and *Coverage*. These evaluation metrics measure the accuracy of protein function prediction according to different aspects, and they have been applied to evaluate the results of multi-label learning and protein function prediction [5, 31, 35]. The formal definition of these metrics are provided in Additional file 1. To keep consistency with other evaluation metrics, we use *1-RankLoss* instead of *RankingLoss*. In this way, the higher the value of all the evaluation metrics (excluding *Coverage*), the better the performance is. Since the various metrics capture different aspects of the methods, it is difficult for a single approach to consistently outperform the others across all the evaluation metrics.

### Missing function prediction

In this section, we conduct experiments to investigate the performance of dRW and dRW-*k*NN on predicting missing functions of partially annotated proteins. For each setting value of *m*, we randomly mask *m* functions of a ‘completely’ annotated protein. The masked functions are considered as missing functions of the protein. We then apply the competing methods to predict the missing functions and evaluate the performance by using the evaluation metrics introduced above. For each setting of *m*, we repeat the mask and evaluation operation in each round for 10 times. In Table 2, we report the average experimental results (with *m*=1,3,5) on proteins of Yeast annotated with BP functions. Other results on Yeast and Human are provided in Tables S1-S5 of Additional file 1. In these tables, the results in **bold** font are the best (or comparable best) statistically significant results, according to a pairwise *t*-test at 95 % significance level.

**Table 2:** Results of predicting the missing *BP* functions of partially annotated *Yeast* proteins (*N*=5914, $|\mathcal {T}|=2979$)

Metric	*m*	dRW-*k*NN	dRW	ITSS	PILL	Naive
MacroF1	1	93.14 ± 0.13	**93.61** ± 0.05	91.66 ± 0.09	91.52 ± 0.15	1.99 ± 0.00
	3	82.72 ± 0.25	**83.29** ± 0.13	80.14 ± 0.14	79.77 ± 0.16	2.01 ± 0.00
	5	74.67 ± 0.22	**75.92** ± 0.17	71.16 ± 0.33	70.96 ± 0.22	2.03 ± 0.00
AvgROC	1	99.88 ± 0.01	**99.93** ± 0.00	98.24 ± 0.02	98.77 ± 0.03	45.88 ± 0.00
	3	**99.55** ± 0.02	**99.59** ± 0.02	94.44 ± 0.08	96.36 ± 0.15	45.88 ± 0.00
	5	**99.01** ± 0.02	98.89 ± 0.03	90.48 ± 0.17	93.83 ± 0.06	45.88 ± 0.00
1-RankLoss	1	99.96 ± 0.00	**99.97** ± 0.00	98.99 ± 0.02	99.81 ± 0.01	91.13 ± 0.00
	3	**99.47** ± 0.03	99.17 ± 0.03	96.89 ± 0.05	99.23 ± 0.03	91.04 ± 0.00
	5	**98.20** ± 0.02	97.63 ± 0.03	93.99 ± 0.10	98.42 ± 0.05	90.95 ± 0.01
Fmax	1	97.97 ± 0.00	**98.08** ± 0.00	97.90 ± 0.00	97.91 ± 0.00	36.96 ± 0.00
	3	**93.99** ± 0.02	93.92 ± 0.01	93.66 ± 0.02	93.61 ± 0.00	36.86 ± 0.00
	5	**90.25** ± 0.03	89.88 ± 0.00	89.66 ± 0.02	89.41 ± 0.00	36.84 ± 0.03
RAccuracy	1	38.75 ± 0.66	**44.68** ± 0.21	12.41 ± 0.48	21.65 ± 0.37	37.51 ± 0.94
	3	**39.75** ± 0.13	36.08 ± 0.24	23.02 ± 0.06	22.27 ± 0.37	37.84 ± 0.75
	5	**40.13** ± 0.43	33.58 ± 0.24	27.39 ± 0.29	23.92 ± 0.08	37.69 ± 0.37
Coverage *↓*	1	78.24 ± 0.95	**66.34** ± 1.10	405.01 ± 9.56	232.52 ± 4.58	1585.06 ± 0.99
	3	**191.34** ± 4.88	234.61 ± 4.92	943.54 ± 10.84	524.50 ± 12.33	1605.22 ± 0.95
	5	**340.09** ± 4.55	469.13 ± 9.17	1412.81 ± 9.85	806.23 ± 18.18	1625.35 ± 3.17

The numbers in boldface denote the best (or comparable best) statistically significant performance (according to a *t*-test at 95 % significance level). *↓* means the lower the value, the better the performance. *m* is the number of missing functions for a protein, *N*_*m*_ is the total number of missing functions, and $|\mathcal {T}_{m}^{0}|$ is the number of the second kind of missing functions of *N* proteins for a given *m*. *m*=1, $|\mathcal {T}_{1}^{0}|=25$, *N*_1_=4705; *m*=3, $|\mathcal {T}_{3}^{0}|=106$, *N*_3_=14079; *m*=5, $|\mathcal {T}_{5}^{0}|=209$, *N*_5_=23299

From these tables, we can observe that dRW-*k*NN and dRW achieve the best results in most cases, and dRW-*k*NN often gets better results than dRW. In summary, out of 108 configurations (2 datasets × 3 GO sub-ontology × 3 settings of *m* × 6 evaluation metrics), dRW-*k*NN outperforms dRW in 65.74 % of the cases, outperforms ITSS in 94.44 % of the cases, and outperforms PILL in 83.33 % of the cases; ties with them in 7.41 %, 4.63 %, and 5.56 % of the cases; and loses to them in 26.85 %, 0.93 %, and 11.11 % of the cases, respectively. dRW-*k*NN also outperforms the baseline approach Naive in almost all the cases. For example, on *RAccuracy* which evaluates how many missing functions of *N* proteins correctly replenished, dRW-*k*NN achieves 12.23 %, 44.11 %, 35.69 % and 105.37 % improvements over dRW, ITSS, PILL and Naive, respectively. The superior results achieved by dRW-*k*NN confirm its effectiveness in predicting missing functions of partially annotated proteins, and also further support the integration of downward random walks with the semantic similarity between proteins for missing function prediction.

dRW directly predicts functions of a protein by performing random walks on the GO DAG, and sometimes achieves comparable results with dRW-*k*NN. From the tables, we can observe that both dRW and dRW-*k*NN statistical significantly outperform ITSS in most cases, the reason is that the former two methods can pre-estimate the likelihood of both kinds of missing functions: the ones that exist in the neighborhood of a protein, and the ones that are not associated with any proteins but are semantically similar to some of the existing ones. In contrast, ITSS can only predict the first kind of missing functions. PILL utilizes Lin’s corpus similarity, which computes the information content of a term based on the term’s frequency in the corpus. For a GO term that does not exist in the corpus, its similarity with respect to other GO terms is set to 0. Therefore, PILL can only predict the first kind of missing functions and it loses to dRW in most configurations.

The Naive method predicts functions based on the frequency of the GO terms of the *N* proteins. It often achieves the lowest performance, and sometimes stable results with respect to some evaluation metrics (i.e., *MacroF1*, *AvgROC*). This fact shows the need of designing tools to effectively predict protein functions. Naive sometimes achieves higher *1-RankLoss* than other methods; this is because *1-RankLoss* favors the predictor that produces correctly ranked pairs of functions, and it is in tune with the Naive method, which ranks the functions based on their frequencies.

The main difference between dRW-*k*NN and ITSS is that dRW-*k*NN takes advantage of dRW to pre-estimate the missing functions, whereas ITSS does not. The performance margin between dRW-*k*NN and ITSS is much larger than the margin between dRW-*k*NN and dRW. This fact shows the downward random walks contribute much more than ITSS (or *k*NN) on predicting missing functions. This observation also demonstrates that downward random walks are of paramount importance to enhance the performance of missing function prediction.

**Fig. 2:**
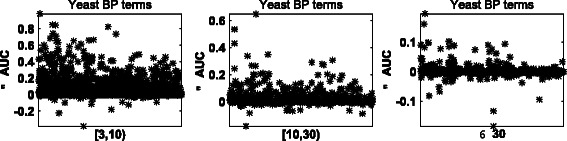
AUC difference between dRW-*k*NN and ITSS. The AUC (Area Under the ROC Curve) difference between dRW-*k*NN and ITSS on proteins of *Yeast* annotated with *BP* terms of different sizes. [3,10) includes 1350 terms, [10,30) includes 761 terms, and ≥30 includes 868 terms

To further study the difference between dRW-*k*NN and ITSS, we measure the Area Under the ROC Curve (AUC) of each GO term, and use *Δ*AUC to represent the AUC difference between dRW-*k*RW and ITSS. To investigate the performance of dRW-*k*NN and ITSS for different levels of sparsity, terms are divided into three groups: (i) terms associated with at least 3 but less than 10 proteins ([3,10)), (ii) terms associated with at least 10 but less than 30 proteins ([10,30)), (iii) and terms associated with at least 30 proteins (≥30). The *Δ*AUC on Yeast annotated with BP terms for each group are reported in Fig. 2. Other results are provided in Fig. S2-S6 of Additional file 1.

From these figures, we can observe that dRW-*k*NN achieves a larger AUC than ITSS with respect to most GO terms. The terms in the group [ 3,10) have the largest *Δ*AUC, followed by the group [ 10,30) and then by the group ≥30. This observation shows the proposed dRW-*k*NN can achieve better results than ITSS on the sparse terms. There are two reasons: (i) dRW-*k*NN applies dRW to pre-estimate the likelihoods of missing functions of a protein, and then makes the prediction based on the pre-estimated likelihoods and on the available functions associated with the neighborhood proteins, while ITSS makes predictions only based on the functions associated with the neighborhood proteins; (ii) dRW-*k*NN can predict the second kind of missing functions, while ITSS can not. For the terms associated with at least 30 proteins, dRW-*k*NN still outperforms ITSS in some cases. These results also support the effectiveness of dRW in estimating missing functions of a partially annotated protein.

### The influence of semantic similarity

We conduct additional experiments to study the influence of dRW based on different semantic similarities between GO terms. Here, we introduce three variants of dRW: dRW-Corpus, dRW-Disjoint and dRW-E. dRW-Corpus performs dRW on the GO hierarchy based on Lin’s corpus similarity, which computes *I* *C*(*t*) by the frequency of term *t* in the corpus. dRW-Disjoint does dRW on the GO hierarchy based on a recently proposed disjointness axioms similarity [41]. dRW-E assumes the downward transition probabilities from a term to its children terms are all equal, and applies the same downward random walks with restart procedure as dRW. The other settings of these methods are kept the same as in the previous experiments. The results on Yeast annotated with BP terms are reported in Table 3. Other results are provided in Tables S6-S10 of Additional file 1. In these tables, the results in **bold** font are the best statistically significant results, according to a pairwise *t*-test at 95 % significance level.

**Table 3:** Results of dRW, dRW-Corpus, dRW-Disjoint, dRW-E in predicting the missing *BP* functions of *Yeast* proteins, $|\mathcal {T}|=2979$ with *m*=3

Metric	dRW	dRW-Corpus	dRW-Disjoint	dRW-E
MacroF1	83.29 ± 0.13	79.77 ± 0.09	83.19 ± 0.09	83.17 ± 0.07
AvgROC	99.59 ± 0.02	93.61 ± 0.07	99.57 ± 0.01	99.57 ± 0.00
1-RankLoss	**99.17 ± 0.03**	93.87 ± 0.05	**99.11 ± 0.02**	98.87 ± 0.01
Fmax	93.92 ± 0.01	93.67 ± 0.00	93.90 ± 0.01	93.89 ± 0.01
RAccuracy	**36.08 ± 0.24**	15.58 ± 0.31	33.65 ± 0.27	32.67 ± 0.50
Coverage *↓*	**234.61 ± 4.92**	1843.95 ± 18.70	242.53 ± 1.44	255.14 ± 5.49

From these tables we can see that dRW achieves better results than the three variants in most cases. Our proposed dRW almost always significantly outperforms dRW-Corpus. The reason is that the GO annotations of proteins are far from complete, dRW-Corpus makes twice use of the annotations, whereas our proposed dRW makes one use of the annotations. Therefore, the latter one is less suffered from the incomplete annotations. There is no explicit disjoint relationship between terms in the GO hierarchy. For the experiments, two terms never co-associated with the same protein are viewed as disjoint terms. For this reason, there are some false disjoint terms. Thus, dRW often performs better than dRW-Disjoint.

dRW performs better than dRW-E in most cases. This fact suggests that the transitional probabilities from a term to its children terms should not be simply treated all equal. The performance margin between dRW and dRW-E are not so obvious as the margin between dRW and dRW-Corpus. A possible reason is that a term may have one child term, and in this case the transitional probability is more determined by *η* than by *W* (see Eq. (4)). In fact, Lin’s structure similarity is also inclined to set equal transitional probabilities. We observe that more than hundreds (or thousands) of terms were used in the experiments, and a small performance margin still means significant improvement. How to achieve improved transitional probability estimation is an important future direction to pursue.

### Historical rollback experiments

To further study dRW, dRW-*k*NN and other comparing methods in situations that reflect real life scenarios, we predict missing BP functions of partially annotated proteins using an older version GOA file (date: 2010-01-20) of Yeast and Human, and then validate the predicted missing functions using a recent GOA file (date: 2014-06-09). The parameters settings of these comparing methods are kept the same as in the previous experiments. We process the older GOA file in the same way as the recent GOA file used in the previous experiments. We keep the root BP GO term (‘GO:0008150’) in the historical rollback experiments, since some new annotations of proteins correspond to the direct child nodes of the root node. After preprocessing, there are 4,338 terms associated with at least one protein in the recent Yeast GOA file. Among these 4,338 terms, 3,581 terms are associated with at least one protein in the older Yeast GOA file. Thus, 757 terms are not associated with any proteins in the older Yeast GOA file. As to Human proteins, there are 11,212 GO terms associated with at least one protein in the recent GOA file. Among these 11,212 terms, only 6,823 terms are associated with at least one protein in the older Human GOA file. Therefore, 4,389 terms are not associated with any protein in the older Human GOA file. Obviously, the historical rollback experiment is more challenging than our previous experiments. ITSS, PILL and Naive can not predict the GO terms that are not associated with any proteins in the older GOA files.

We choose the top 100 largest likelihoods predicted by each of the comparing methods and report the number of true positive predictions in Table 4. The detailed information of these positive predictions and the ones augmented by the true path rule on these 100 predictions are listed in four excel sheet files (see Additional files 2–5). To save space, we just list the true positive predictions made by dRW on Yeast and Human in Table 5. From the results in Tables 4, 5 and positive predictions in excel files, we have some interesting observations.

**Table 4:** Numbers of true positive predictions made by dRW, dRW-*k*NN, ITSS, PILL and Naive from an older GOA file (date: 2010-01-20) to a recent GOA file (date: 2014-06-09) of Yeast and Human. The data in the parentheses are the corresponding true positive rate for each of the methods. TPR means the true path rule is applied to append the ancestor functions of the positive predictions, and NoTPR means the true path rule is not applied

		dRW	dRW-*k*NN	ITSS	PILL	Naive
Yeast	NoTPR	6(6.00 %)	17(17.00 %)	6(6.00 %)	0(0.00 %)	31(31.00 %)
	TPR	34(6.58 %)	17(17.00 %)	6(6.00 %)	11(1.83 %)	31(31.00 %)
Human	NoTPR	10(10.00 %)	27(27.00 %)	20(20.00 %)	19(19.00 %)	48(48.00 %)
	TPR	120(17.36 %)	27(27.00 %)	20(20.00 %)	80(21.45 %)	48(48.00 %)

**Table 5:** Examples of correctly predicted missing BP functions by dRW from an older GOA file (date: 2010-01-20) to a recent GOA file (date: 2014-06-09) of Yeast and Human. hCount gives the number of proteins annotated with the term in the older GOA file. Depth represents the term’s depth in the GO hierarchy

Yeast				Human			
Protein	GO terms	hCount	Depth	Protein	GO terms	hCount	Depth
HIS5	GO:0001193	0	8	EZR	GO:0002143	0	9
PET494	GO:0019379	0	5	TMEM200C	GO:0007094	8	8
CYC1	GO:0019430	0	4	C9orf96	GO:0016056	3	7
FES1	GO:0044718	0	5	DRGX	GO:0035511	0	5
MET17	GO:0090334	0	6	HDAC7	GO:0035511	0	5
TSTA3	GO:2000679	2	7	QPRT	GO:0045040	0	5
				CSHL1	GO:0045040	0	5
				RGS3	GO:0060397	2	7
				PRDM7_V2	GO:0071300	0	6
				TMEM82	GO:0090050	2	7

The true positive predictions made by dRW are different from those of other comparing methods. Both dRW and dRW-*k*NN take advantage of downward random walks, but they do not share any true positive predictions. The reason is that dRW directly uses the available annotations of a partially annotated protein and GO hierarchy to replenish the missing functions, and dRW-*k*NN replenishes the missing functions of a protein based on the GO annotations associated with its neighbors. dRW and PILL make different true positive predictions. The cause is that PILL utilizes Lin’s corpus similarity and it does not utilize the GO structure in replenishing the missing functions. Both dRW-*k*NN and ITSS predict the missing functions of a protein by the GO annotations associated with its neighbors, they share 2 true positive predictions on Yeast and 14 true positive predictions on Human. For example, they correctly predict that SAC7 (a protein of Yeast) is annotated with GO:0006259 and GO:0044260.

The false positive predictions made by dRW and those of other comparing methods are also quite different. For example, dRW wrongly predicts that Yeast protein ENO2 is annotated with GO:0034462, whereas dRW-*k*NN wrongly predicts that ENO2 is annotated with GO:0000027 and GO:0042273. That is because dRW gives high priority to a GO term that has large semantic similarity with the terms associated with a protein, and dRW-*k*NN prefers terms associated with the neighbors of the protein. dRW and PILL do not share any false positive predictions. For example, dRW assigns GO: 0071507 to Yeast proteins ATP6 and CHO2, and PILL assigns GO:0071507 to Yeast protein MDL1. They also wrongly assign different GO terms to the same protein. dRW assigns GO:0043001 to Yeast protein CPA1, whereas PILL assigns GO:0030476 and GO:0070591 to CPA1. dRW-*k*NN and ITSS share 30 false positive predictions on Human and they share 10 false positive predictions on Yeast proteins PTP1, SIP18, SNU13 and YSF13, but they assign different terms to protein PTP1. These facts can be attributed to that dRW-*k*NN replenishes the functions by the functions associated with the neighbors of a protein, and the ones pre-estimated by dRW, whereas ITSS only uses the functions associated with the neighbors of the protein.

Regardless of the application of the true path rule, the positive predictions produced by dRW-*k*NN, ITSS and Naive remain the same. By applying the true path rule, the number of positive predictions produced by dRW and PILL increases sharply, and dRW achieves the largest number of true positive predictions. dRW can predict the first and second kind of missing functions, and thus for dRW-*k*NN. In contrast, the other comparing methods can only predict the first kind of missing functions. The missing functions predicted by dRW are not only sparse (associated with no more than 10 proteins), but also locating at relatively deeper levels (≥4) in the GO hierarchy. These results indicate that dRW can more accurately predict functions that locate in deep levels of the GO hierarchy than others, and also support our motivation to apply dRW along with the GO hierarchy for missing function prediction.

As to the true positive predictions made by Naive, they are always associated with the same GO term (‘GO:0009987’ for Yeast proteins and ‘GO:0044699’ for Human proteins). In the older GOA files, these two terms, locating at the 2nd level of the GO hierarchy, are associated with 3768 Yeast proteins and 5517 Human proteins, respectively. Thus, we can say that the functions predicted by Naive produces are rather shallow.

By applying the true path rule, the number of true positive predictions made by PILL increases from 0 to 11 (on Yeast) and from 19 to 80 (on Human). PILL directly uses the available functions of a protein and the semantic similarity between GO terms to predict the missing functions of proteins, but it only uses the GO hierarchy to compute the semantic similarity between terms, and does not use the hierarchy in the process of missing function prediction. In addition, the semantic similarity between terms is calculated based on Lin’s corpus similarity. Therefore, PILL loses to dRW and it can not predict the second kind of missing functions.

dRW-*k*NN gets a larger number of true positive predictions than ITSS, although they both utilize the same semantic similarity between proteins and a *k*NN style classifier to predict missing functions of proteins. The cause is that dRW-*k*NN takes advantage of dRW to pre-estimate the missing functions. The difference between dRW-*k*NN and ITSS supports the benefit of using dRW for missing functions prediction. However, after applying the true path rule on the top 100 positive predictions, these two methods do not produce any new true positive predictions, whereas dRW makes more true positive predictions. That is because (i) dRW-*k*NN and ITSS predict missing functions of a protein based on the functions associated to its neighborhood proteins, and the larger the frequency of a function is, the more likely it is considered as missing for the protein; (ii) the probability of functions associated with neighbors is set to 1, and the probability of missing functions of a protein pre-estimated by dRW is smaller than 1. Since we only choose the top 100 predictions, the functions associated with neighbors are favored against the pre-estimated ones. From these examples, we can see that dRW is inclined to predicting the second kind of missing functions and dRW-*k*NN is biased towards the prediction of the first kind of missing functions.

Even if applied the true path rule, the true positive rates achieved by dRW on Yeast and Human, and that made by dRW-*k*NN are lower than that produced by Naive. However, we should not simply conclude that Naive performs better than the former two methods. From the list of true positive predictions in the four additional excel files, the missing functions of proteins predicted by Naive are shallow and bring little biological knowledge, these functions are associated with a large number of proteins and locates at the 2nd level of GO hierarchy. In contrast, the missing functions of proteins predicted by dRW and dRW-*k*NN are not only locating at deeper levels than the ones made by Naive, but also associated with much fewer proteins in the older GOA files.

dRW and dRW-*k*NN achieve lower accuracy in the rollback experiments than in the masked GO terms experiments. The reasons are three fold: (i) both the GO structure and the terms are updated from 2010 to 2014. For example, the number of direct GO annotations (without appending the ancestor terms via true path rule) provided in the Human GOA file increases from 29,407 to 74,109 by 2014-06-09, the number of terms in GO also increases from 34,427 to 41,239. (ii) The number of considered GO terms in the historical rollback are 4,338 for Yeast and 11,212 for Human, whereas the number of considered terms in the previous experiments are 2,979 and 7,294, respectively. In addition, the number of second kind of missing functions in the masked GO terms experiments are about 209 for Yeast and 135 for Human, and the number of second kind of missing functions in the rollback experiments are 757 for Yeast and 4,389 for Human. (iii) The masked GO terms experiments randomly mask some leaf terms and treat these masked terms as missing functions of proteins, but the appended missing functions of a protein do not always follow the same random pattern. In fact, in the recent GOA file, we found that the appended missing functions of a protein are the descendants of one or several terms associated with the protein, instead of all the terms associated with the protein. For example, the appended annotations of Human protein ALG6 are the descendants of GO:0006464, and the appended annotations of Human protein CLDN16 are descendants of GO:0008150.

In the end, we have to keep in mind that the number of true positive predictions is *conservative*, since a positive prediction without a corresponding validated annotation might simply indicate a lack of study of the protein, rather than an incorrect prediction. The proteins in the recent GOA file are still partially annotated. Over time, more true positive predictions will be validated and also some false negative predictions may be resulted in. We also have to notice that dRW, dRW-*k*NN and the comparing methods can bring over-annotated functions of proteins, and they are not the best approach for every protein. These over-annotated functions of a protein are descendants of the available functions of the protein, they are often corresponding to specific ones. In addition, these over-annotated functions do not have biological evidence support. The reason is that dRW and dRW-*k*NN only use the GO structure and available annotations of proteins to predict the missing functions. How to address these limitations is an open problem and interesting to pursue in future work. One possible way is to filter out the over-annotated functions by referring to other data sources (i.e., biomedical text and the text description of ontological terms) and softwares (i.e., GOAnnotator [36]). We still believe our work can drive more research on predicting missing functions of partially annotated proteins. These missing functions often bring much more biological information and are more interested to biologist than the available annotations of proteins. Over all, these historical rollback experiments verify the ability of dRW and dRW-*k*NN in predicting missing functions of partially annotated proteins.

## Conclusions and future work

In this paper we study the problem of predicting new functions for partially annotated proteins. We propose two methods, dRW and dRW-*k*NN, that perform downward random walks with restart on the Gene Ontology directed acyclic graph, and the available functions of proteins to predict missing ones. The proposed models are able to predict two kinds of missing functions: the functions that are associated with some proteins but are missing for others; and the ones missed for all the proteins of interest but that exist in the GO hierarchy. Our empirical study on the proteins of Yeast and Human shows that the proposed models outperform several competitive related methods. This paper will drive more research on missing function prediction of partially annotated proteins. As part of our future work, we are interested in investigating other semantic similarities between GO terms and incorporating protein specific information to accurately predict missing functions.

## Endnotes

^1^ http://geneontology.org/page/download-ontology. (accessed: 2014, July 1st)

^2^ ftp://ftp.ebi.ac.uk/pub/databases/GO/goa/. (accessed: 2014, June 9th)

^3^ http://geneontology.org/page/go-annotation-conventions. (accessed: 2014, July 1st)

## Additional files

Additional file 1
**Supplementary file of ‘Predicting protein function via downward random walks on a gene ontology’.** This PDF file includes examples of missing functions, parameters setting, definition of evaluation metrics, and additional experimental results. (PDF 495 kb)

Additional file 2
**Yeast_bp(NoTPR).** This Excel Spreadsheet file includes true positive predictions, false positive predictions of the top 100 predictions made by dRW, dRW-*k*NN, ITSS, PILL and Naive on *Yeast* proteins annotated with Biological Process Ontology. (XLS 73 kb)

Additional file 3
**Yeast_bp(TPR).** This Excel Spreadsheet file includes true positive predictions, false positive predictions augmented by the True Path Rule on the top 100 positive predictions made by dRW, dRW-*k*NN, ITSS, PILL and Naive on *Yeast* proteins annotated with Biological Process Ontology. (XLS 148 kb)

Additional file 4
**Human_bp(NoTPR).** This Excel Spreadsheet file includes true positive predictions, false positive predictions of the top 100 predictions made by dRW, dRW-*k*NN, ITSS, PILL and Naive on *Human* proteins annotated with Biological Process Ontology. (XLS 132 kb)

Additional file 5
**Human_bp(TPR).** This Excel Spreadsheet file includes true positive predictions, false positive predictions augmented by the True Path Rule on the top 100 positive predictions made by dRW, dRW-*k*NN, ITSS, PILL and Naive on *Human* proteins annotated with Biological Process Ontology. (XLS 242 kb)
