# Supplementary material for: Predicting protein function via downward random walks on a gene ontology
Source: BMC Bioinformatics. 2015 Aug 27;16:271. doi: 10.1186/s12859-015-0713-y (PMC4551531; doi:10.1186/s12859-015-0713-y)
Supplement: Additional file 1 — Supplementary file of ‘Predicting protein function via downward random walks on a gene ontology’. This PDF file includes examples of missing functions, parameters setting, definition of evaluation metrics, and additional experimental results. (PDF 495 kb) [file 12859_2015_713_MOESM1_ESM.pdf]

Supplementary file of ‘Prediction protein functions via downward random walks on a gene ontology’

Guoxian Yu[1,2,*], Hailong Zhu[3,*], Carlotta Domeniconi[4] and Jiming Liu[3]

[1]College of Computer and Information Sciences, Southwest University, Chongqing, China

[2] Key Laboratory of Symbolic Computation and Knowledge Engineering of Ministry of Education, Jilin University, Changchun 130012, China.

[3] Department of Computer Science, Hong Kong Baptist University, Hong Kong

[4]Department of Computer Science, George Mason University, VA, USA

[*]Contact: gxyu@swu.edu.cn; hlzhu@comp.hkbu.edu.hk

August 2, 2015

1 Examples of Missing Functions

To better understand the pattern of missing functions of a protein, we separately illustrate the BP GO annotations of Human proteins ALG6 and CLDN16 from an old GO annotation (GOA) file to a recent GOA file in Figure S1. In the figure, GO terms in the yellow boxes not circled by blue eclipses are the available GO annotations of the protein by 2010-01-20, and the GO terms in the yellow box circled by blue eclipses are the appended GO annotations of the protein by 2014-06-09. These appended functions are the missing functions of the protein. From the figure, it is easy to find that the missing functions of a partially annotated protein are the descendants of the terms that already associated with the protein.

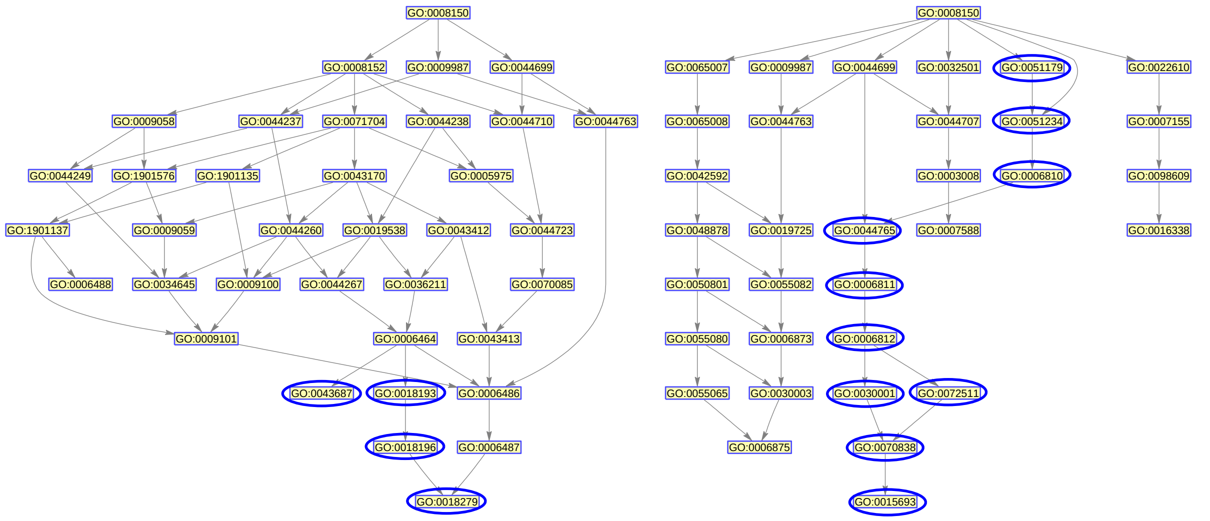

(a) GO annotations of ALG6 (b) GO annotations of CLDN16

Figure S1: For each sub-figure, the BP GO terms in the yellow boxes are the available GO annotations of the protein by 2010-01-20, and BP GO terms circled by blue eclipses are the appended annotations of the protein by 2014-06-09.

2 Parameter setting and evaluation metrics

2.1 Parameter setting

For the parameter setting of dRW and dRW-kNN, we simply set $\eta = 0.5$ (see Eq. (4) in the main text), which means that a random walker has an equal probability of staying at the start node or to reach its descendant nodes. We optimized the neighborhood size k in $\{1, 2, 3, 4, 5, 10, 20, 30, 40, 50\}$ by five-fold cross validation under the setting of $m = 3$. We then chose the parameter values that produced the best results. For the experiments on Yeast, we set $k = 10$, and for the experiments on Human, we set

$k = 50$. ITSS needs to set the neighborhood size k and the threshold value (see Eq. (3) in [1]). The threshold is used to remove pairs of GO terms with low similarity and thus to reduce noise in computing the semantic similarity between proteins. We optimized k in the same range as dRW-kNN and optimized the threshold value from 0 to 1 with step-size 0.1 by five-fold cross validation as for dRW-kNN. For both experiments on Yeast and Human, we set $k = 50$ and the threshold value to 0.9. In the experiments, PILL utilizes the correlation between pairwise function categories to estimate missing functions, without using the protein-protein interactions as in the original paper. In fact, PILL can obtain similar results by directly using the function correlations even without the protein-protein interactions. As the authors reported in [2], we set the threshold value for correlations between functions as 0.05. Naive does not need to set any parameter, since it predicts protein functions simply based on the frequency of functions.

2.2 Evaluation metrics

Here, we give the formal definition of the six evaluation metrics introduced in the main tex: *MacroF1*, *AvgROC*, *RankingLoss*, *Fmax*, *RAccuracy*, and *Coverage*. These metrics are widely used in multi-label learning and protein function prediction [3, 4, 2, 5].

MacroF1 is the average F measure of different GO terms:

$$MacroF1 = \frac{1}{|\mathcal{T}|}\sum_{t\in\mathcal{T}}\frac{2p_t \times r_t}{p_t + r_t}$$

where $|\mathcal{T}|$ is the total number of distinct GO terms (each term corresponds to a label) in $\mathcal{T}$, p_t and r_t are the precision and recall of the t-th term, defined as:

$$p_t = \frac{TP_t}{TP_t + FP_t}, \quad r_t = \frac{TP_t}{TP_t + FN_t}$$

TP_t, FP_t, and FN_t are the number of true positive predictions, false positive predictions, and false negative predictions with respect to the t-th term. From the definition, it can be observed *MacroF1* first calculates the F measure for each term, and then averages over all the GO terms. *MacroF1* is more affected by the performance on sparse terms that associate with fewer proteins, and the missing functions of a protein often correspond to sparse terms. For this reason, we choose *MacroF1* as an evaluation metric.

Average ROC (AvgROC) score is a function centric evaluation metric, it averages the receiver operation curve (ROC) score of each function. The ROC score is calculated as the area under the ROC curve, which plots the true positive rate (sensitivity) as a function of the false positive rate (1-specificity) under different classification thresholds. It measures the overall quality of the ranking induced by the classifier, instead of the quality of a single value of the threshold in that ranking.

Ranking loss evaluates the average fraction of GO term pairs that are not correctly ranked:

$$RankingLoss = \frac{1}{N}\sum_{i=l}^{N}\frac{1}{|\mathcal{T}_i||\bar{\mathcal{T}}_i|}|\{(t_1,t_2) \in \mathcal{T}_i \times \bar{\mathcal{T}}_i | \mathcal{L}(i,t_1) \leq \mathcal{L}(i,t_2)\}|$$

where $\mathcal{T}_i$ is the GO annotations of protein i, and $\bar{\mathcal{T}}_i$ is the complement set of $\mathcal{T}_i$; $\mathcal{L}(i,t)$ is the predicted likelihood for the i-th protein annotated with the term t. The smaller the value of *RankLoss*, the better the performance is.

Fmax is a protein centric evaluation metric suggested in the community-based critical assessment of protein function annotation (CAFA) [4]. *Fmax* is an F-measure defined as:

$$Fmax = \max_{\tau\in[0,1]}\frac{2p(\tau) \times r(\tau)}{p(\tau) + r(\tau)}$$

where $p(\tau) = \frac{1}{m(\tau)}\sum_{i=1}^{m(\tau)} p_i(\tau)$ is the precision at threshold τ, $p_i(\tau)$ is the precision on the i-th protein, $m(\tau)$ is the number of proteins on which at least one prediction was made above the threshold τ, $r(\tau) = \frac{1}{N}\sum_{i=1}^{N} r_i(\tau)$ is the recall across N proteins at threshold τ.

RAccuracy evaluates, overall, how many missing functions of N proteins are correctly replenished:

$$RAccuracy = \frac{1}{N}\sum_{i=1}^{N}\frac{|(\mathcal{T}_i - \hat{\mathcal{T}}_i) \cap \tilde{\mathcal{T}}_i|}{|\mathcal{T}_i - \hat{\mathcal{T}}_i|}$$

where $\hat{\mathcal{T}}_i$ represents the initial functions associated with the i-th partially annotated protein, $\tilde{\mathcal{T}}_i$ represents the predicted missing functions, $(\mathcal{T}_i - \hat{\mathcal{T}}_i)$ corresponds to the missing functions, and $(\mathcal{T}_i - \hat{\mathcal{T}}_i) \cap \tilde{\mathcal{T}}_i$ contains the correctly predicted missing functions for this protein.

Coverage evaluates how far, on average, we need to go down the GO terms ranking list to cover all the ground-truth annotations of the protein:

$$Coverage = \frac{1}{N}\sum_{i=1}^{N} \max_{t\in\mathcal{T}_i} rank(\mathcal{L}(i,t)) - 1$$

where $rank(\mathcal{L}(i,\cdot))$ is a rank function, which ranks the largest $\mathcal{L}(i,\cdot) \in R^{|\mathcal{T}|}$ as 1 and the smallest $\mathcal{L}(i,\cdot)$ as $|\mathcal{T}|$. *Coverage* is often bigger than 1. Obviously, the smaller the value of *Coverage* is, the better the performance is.

To keep consistency with other evaluation metrics, we report *1-RankLoss* instead of *RankLoss*. Thus, the higher the value of these evaluation metrics (except *Coverage*), the better the performance is. *MacroF1* and *RAccuracy* require the predicted likelihood score vector $\mathcal{L}(i,\cdot)$ to be a binary indicator vector. We consider the functions corresponding to the q largest values of $\mathcal{L}(i,\cdot)$ as the functions of the i-th protein. In the experiments, $q = |\mathcal{T}_i|$.

3 Missing function prediction

In the main text, we reported the results on the Yeast annotated with BP functions. Here, the results on Yeast and Human annotated with functions in other sub-ontologies are provided in Tables S1-S5. The results of the AUC difference between dRW-kNN and ITSS on different groups of GO terms on Yeast and Human are reported in Figures S2-S6. Obviously, these results lead to similar observations and conclusions as in the main text.

Table S1: Results of predicting the missing *CC* functions of partially annotated *Yeast* proteins ($N = 5914$, $|\mathcal{T}| = 731$). The numbers in **boldface** denote the best (or comparable best) statistically significant performance (according to a t-test at 95% significance level). ↓ means the lower the value, the better the performance. m is the number of missing functions for a protein, N_m is the total number of missing functions and $|\mathcal{T}_m^0|$ is the number of the second kind of missing functions of N proteins for a given m. $m = 1$, $|\mathcal{T}_1^0| = 51$, $N_1 = 4107$; $m = 3$, $|\mathcal{T}_3^0| = 150$, $N_3 = 12293$; $m = 5$, $|\mathcal{T}_5^0| = 238$, $N_5 = 20141$.

Metric	m	dRW-kNN	dRW	ITSS	PILL	Naive
MacroF1	1	80.56±0.48	**82.45±0.38**	75.65±0.42	76.59±0.33	3.01±0.00
	3	60.50±0.55	**61.23±0.64**	52.29±0.37	52.61±0.38	3.01±0.02
	5	46.51±0.31	**48.71±0.48**	38.95±0.38	38.78±0.45	3.00±0.00
AvgROC	1	**99.40±0.03**	**99.40±0.06**	93.39±0.28	94.86±0.13	49.27±0.00
	3	**96.64±0.12**	96.31±0.07	82.93±0.35	86.33±0.46	49.27±0.00
	5	**92.76±0.15**	92.10±0.30	75.13±0.48	78.79±0.27	49.27±0.00
1-RankLoss	1	**99.83±0.01**	**99.83±0.01**	96.30±0.06	99.22±0.07	94.94±0.01
	3	**97.96±0.03**	97.64±0.03	87.61±0.12	**97.93±0.07**	94.55±0.03
	5	94.94±0.04	94.20±0.03	78.30±0.14	**95.41±0.04**	94.11±0.05
Fmax	1	96.44±0.01	**96.54±0.00**	96.26±0.00	96.28±0.02	63.53±0.00
	3	**89.82±0.08**	89.45±0.06	88.59±0.02	88.03±0.01	63.04±0.00
	5	**83.85±0.07**	83.01±0.04	82.06±0.07	79.70±0.03	62.75±0.00
RAccuracy	1	**38.56±0.59**	36.16±0.33	19.38±1.44	7.21±0.54	3.26±0.21
	3	**44.72±0.55**	38.21±0.51	31.95±0.62	17.04±0.09	15.14±0.39
	5	**44.00±0.16**	37.89±0.18	35.38±0.31	28.74±0.21	23.00±0.12
Coverage ↓	1	**33.06±0.62**	34.52±0.38	215.20±4.93	105.48±7.90	303.77±0.89
	3	**113.54±1.29**	131.06±0.68	383.23±5.53	190.79±9.90	319.75±3.49
	5	**210.32±1.74**	242.81±1.07	497.65±3.11	293.85±3.27	333.52±2.10

4 The Influence of Semantic Similarity

The experimental results of dRW, dRW-Corpus, dRW-Disjoint and dRW-E on Yeast and Human are provided in Tables S6-S10. dRW-Corpus performs downward random walks with restart on the GO hierarchy based on the Lin's corpus similarity [6]. dRW-Disjoint does downward random walks with restart based on the recently proposed disjoint axioms similarity [7]. dRW-E assumes the downward transition probabilities from a GO term to its children GO terms are all equal, and then applies dRW on the GO hierarchy. These data also provide similar observations and conclusions as given in the main text.

Table S2: Results of predicting the missing *MF* functions of partially annotated *Yeast* proteins ($N = 5914$, $|\mathcal{T}| = 546$). The numbers in **boldface** denote the best (or comparable best) statistically significant performance (according to a t-test at 95% significance level). $\downarrow$ means the lower the value, the better the performance. m is the number of missing functions for a protein, N_m is the total number of missing functions and $|\mathcal{T}_m^0|$ is the number of the second kind of missing functions of N proteins for a given m. $m = 1$, $|\mathcal{T}_1^0| = 71$, $N_1 = 4304$; $m = 3$, $|\mathcal{T}_3^0| = 246$, $N_3 = 11362$; $m = 5$, $|\mathcal{T}_5^0| = 386$, $N_5 = 16829$.

Metric	m	dRW-kNN	dRW	ITSS	PILL	Naive
	1	78.84±0.31	**81.22±0.18**	74.65±0.39	75.15±0.34	1.20±0.01
MacroF1	3	53.35±0.60	**55.71±0.13**	47.25±0.69	47.54±0.70	1.26±0.01
	5	36.72±0.53	**40.48±0.35**	31.97±0.60	31.70±0.15	1.35±0.01
	1	**99.64±0.01**	99.55±0.02	92.50±0.33	94.76±0.07	43.67±0.00
AvgROC	3	**94.08±0.10**	93.33±0.06	77.33±0.41	83.14±0.76	43.67±0.00
	5	**84.20±0.11**	82.71±0.10	67.69±0.59	74.74±0.26	43.67±0.00
	1	**99.71±0.03**	**99.69±0.02**	88.79±0.04	98.36±0.06	90.83±0.02
1-RankLoss	3	92.09±0.09	90.92±0.06	68.96±0.12	**93.05±0.22**	89.43±0.08
	5	80.85±0.08	79.46±0.06	52.61±0.21	**88.49±0.24**	87.40±0.20
	1	89.14±0.04	**89.38±0.01**	88.83±0.00	88.81±0.00	53.07±0.00
Fmax	3	**74.38±0.06**	73.70±0.03	73.78±0.10	73.03±0.23	51.79±2.21
	5	62.97±0.07	61.84±0.04	**63.16±0.09**	60.84±0.16	40.06±0.00
	1	**33.21±0.46**	**31.44±0.42**	22.13±0.61	**32.35±0.98**	14.81±0.61
RAccuracy	3	**31.43±0.48**	26.20±0.10	24.52±0.31	26.88±0.67	20.07±1.74
	5	**28.40±0.31**	24.65±0.05	20.48±0.24	27.09±0.36	16.15±0.10
	1	**18.06±0.79**	**19.39±0.57**	221.70±2.35	85.52±1.22	285.47±0.61
Coverage $\downarrow$	3	**167.82±1.96**	204.25±1.66	517.77±6.47	246.64±6.39	322.78±1.43
	3	365.83±2.50	411.85±1.63	628.04±3.27	**331.91±5.90**	345.65±2.55

Table S3: Results of predicting the missing *BP* functions of partially annotated *Human* proteins ($N = 19009$, $|\mathcal{T}| = 7294$). The numbers in **boldface** denote the best (or comparable best) statistically significant performance (according to a t-test at 95% significance level). $\downarrow$ means the lower the value, the better the performance. m is the number of missing functions for a protein, N_m is the total number of missing functions and $|\mathcal{T}_m^0|$ is the number of the second kind of missing functions of N proteins for a given m. $m = 1$, $|\mathcal{T}_1^0| = 6$, $N_1 = 11899$; $m = 3$, $|\mathcal{T}_3^0| = 55$, $N_3 = 35562$; $m = 5$, $|\mathcal{T}_5^0| = 136$, $N_5 = 58958$.

Metric	m	dRW-kNN	dRW	ITSS	PILL	Naive
	1	**95.81±0.04**	93.90±0.04	**95.65±0.11**	95.08±0.05	1.37±0.00
MacroF1	3	**89.12±0.08**	85.86±0.06	**89.04±0.04**	87.79±0.05	1.38±0.00
	5	**83.64±0.10**	80.08±0.12	**83.60±0.05**	81.61±0.15	1.38±0.00
	1	99.89±0.00	**99.96±0.00**	98.64±0.03	99.39±0.03	47.24±0.00
AvgROC	3	99.63±0.01	**99.78±0.00**	96.15±0.04	98.22±0.01	47.24±0.00
	5	99.27±0.01	**99.45±0.01**	93.65±0.04	96.95±0.03	47.24±0.00
	1	**99.97±0.00**	**99.97±0.00**	99.09±0.03	99.90±0.00	93.90±0.00
1-RankLoss	3	**99.77±0.01**	99.21±0.01	96.65±0.08	99.61±0.01	93.71±0.01
	5	98.99±0.01	97.68±0.03	93.14±0.03	**99.27±0.02**	93.54±0.00
	1	98.00±0.00	**98.04±0.00**	97.98±0.00	97.98±0.00	34.97±0.00
Fmax	3	**94.23±0.01**	94.07±0.01	93.98±0.01	93.97±0.00	34.95±0.00
	5	**90.62±0.03**	90.17±0.01	90.08±0.01	89.98±0.00	34.93±0.00
	1	26.94±0.26	**30.92±0.28**	21.16±0.64	18.81±0.20	1.38±0.10
RAccuracy	3	**29.09±0.19**	28.47±0.21	25.64±0.38	20.98±0.09	3.26±0.03
	5	**30.96±0.19**	27.80±0.16	28.11±0.10	22.27±0.07	4.96±0.07
	1	194.41±3.34	**116.57±2.38**	1023.38±18.36	417.25±6.67	3441.53±1.47
Coverage $\downarrow$	3	**440.72±5.93**	549.45±6.47	2470.33±4.95	913.06±16.15	3656.73±13.07
	5	**797.87±6.64**	1225.37±14.85	3455.13±21.79	1292.92±22.81	3795.66±2.89

References

[1] Tao, Y., Sam, L., Li, J., Friedman, C., Lussier, Y.A.: Information theory applied to the sparse gene ontology annotation network to predict novel gene function. Bioinformatics **23**(13), 529–538 (2007)

[2] Yu, G., Zhu, H., Domeniconi, C.: Predicting protein functions using incomplete hierarchical labels. BMC Bioinformatics **16**(1), 1 (2015)

[3] Zhang, M.-L., Zhou, Z.-H.: A review on multi-label learning algorithms. IEEE Transactions on Knowledge and Data Engineering **26**(8), 1819–1837 (2014)

Table S4: Results of predicting the missing *CC* functions of partially annotated *Human* proteins ($N = 19009$, $|\mathcal{T}| = 978$). The numbers in **boldface** denote the best (or comparable best) statistically significant performance (according to a t-test at 95% significance level). $\downarrow$ means the lower the value, the better the performance. m is the number of missing functions for a protein, N_m is the total number of missing functions and $|\mathcal{T}_m^0|$ is the number of the second kind of missing functions of N proteins for a given m. $m = 1$, $|\mathcal{T}_1^0| = 14$, $N_1 = 11899$; $m = 12375$, $|\mathcal{T}_3^0| = 65$, $N_3 = 36831$; $m = 5$, $|\mathcal{T}_5^0| = 135$, $N_5 = 59773$.

Metric	m	dRW-kNN	dRW	ITSS	PILL	Naive
	1	**85.38±0.35**	83.67±0.08	**85.72±0.20**	**85.12±0.31**	2.50±0.00
MacroF1	3	66.33±0.59	66.47±0.41	65.79±0.32	65.36±0.24	2.50±0.00
	5	52.14±0.42	**55.06±0.28**	52.00±0.67	51.42±0.34	2.50±0.00
	1	99.64±0.02	**99.73±0.01**	96.20±0.12	98.19±0.17	45.25±0.00
AvgROC	3	**98.32±0.06**	98.11±0.06	87.30±0.31	93.72±0.37	45.25±0.00
	5	**96.59±0.12**	95.62±0.09	79.75±0.51	88.64±0.48	45.25±0.00
	1	**99.74±0.01**	99.65±0.01	94.58±0.02	**99.75±0.01**	97.15±0.00
1-RankLoss	3	97.00±0.03	96.36±0.02	83.83±0.09	**99.16±0.02**	97.01±0.00
	5	94.58±0.06	93.23±0.09	74.08±0.13	**98.50±0.02**	96.88±0.00
	1	96.02±0.00	**96.14±0.00**	95.74±0.00	95.74±0.00	56.82±0.00
Fmax	3	**88.19±0.05**	87.38±0.02	87.18±0.05	86.56±0.00	56.21±0.00
	5	**82.23±0.04**	80.81±0.05	79.81±0.05	77.70±0.00	56.21±0.00
	1	**34.88±0.57**	26.65±0.19	22.94±0.28	**33.60±0.82**	7.43±0.17
RAccuracy	3	**40.18±0.19**	31.46±0.11	29.05±0.39	36.20±0.23	16.56±0.10
	5	**42.84±0.18**	33.48±0.22	30.88±0.23	42.14±0.08	23.85±0.16
	1	**42.71±0.72**	55.40±0.44	336.71±1.96	58.65±1.53	218.58±0.13
Coverage $\downarrow$	3	168.92±1.17	221.12±1.63	508.32±2.11	**123.07±3.11**	234.01±0.81
	5	259.65±1.79	347.42±2.56	639.25±1.26	**177.22±1.59**	245.48±1.06

Table S5: Results of predicting the missing *MF* functions of partially annotated *Human* proteins ($N = 5914$, $|\mathcal{T}| = 1772$). The numbers in **boldface** denote the best (or comparable best) statistically significant performance (according to a t-test at 95% significance level). $\downarrow$ means the lower the value, the better the performance. m is the number of missing functions for a protein, N_m is the total number of missing functions and $|\mathcal{T}_m^0|$ is the number of the second kind of missing functions of N proteins for a given m. $m = 1$, $|\mathcal{T}_1^0| = 50$, $N_1 = 11104$; $m = 3$, $|\mathcal{T}_3^0| = 234$, $N_3 = 28533$; $m = 5$, $|\mathcal{T}_5^0| = 421$, $N_5 = 43297$.

Metric	m	dRW-kNN	dRW	ITSS	PILL	Naive
	1	81.32±0.11	**81.76±0.13**	81.30±0.27	80.38±0.08	0.68±0.00
MacroF1	3	57.44±0.37	**60.24±0.16**	57.08±0.18	56.18±0.18	0.72±0.01
	5	41.06±0.28	**46.45±0.27**	41.20±0.34	40.25±0.35	0.77±0.00
	1	**99.58±0.03**	**99.51±0.03**	94.23±0.17	97.23±0.13	45.40±0.00
AvgROC	3	**96.21±0.05**	95.29±0.09	81.39±0.12	89.67±0.37	45.40±0.00
	5	**90.01±0.12**	87.94±0.08	71.52±0.29	81.45±0.40	45.40±0.00
	1	**99.70±0.01**	99.65±0.02	86.41±0.05	99.19±0.01	92.77±0.01
1-RankLoss	3	94.04±0.03	91.93±0.04	70.59±0.08	**96.57±0.05**	92.03±0.00
	5	85.39±0.12	82.41±0.07	55.76±0.06	**93.91±0.07**	91.36±0.04
	1	88.93±0.01	**89.12±0.01**	88.84±0.00	88.83±0.00	42.77±0.00
Fmax	3	**77.48±0.02**	76.73±0.01	76.38±0.07	75.13±0.00	42.77±0.00
	5	**67.05±0.04**	65.92±0.03	65.79±0.04	64.04±0.01	42.77±0.00
	1	**38.37±0.46**	35.60±0.23	19.19±0.36	33.80±1.56	23.12±0.33
RAccuracy	3	**34.78±0.29**	28.29±0.11	23.33±0.10	33.52±0.12	15.19±3.29
	5	**33.50±0.21**	28.06±0.17	19.27±0.10	30.91±0.23	15.52±0.06
	1	**37.04±0.36**	40.84±1.72	468.58±3.81	102.67±1.73	540.37±0.33
Coverage $\downarrow$	3	**238.90±2.76**	364.05±2.79	978.72±5.86	285.53±5.05	585.37±0.89
	5	535.30±4.26	704.74±3.09	1212.38±6.06	**432.70±7.50**	614.82±1.99

[4] Radivojac, P., Clark, W.T., Oron, T.R., Schnoes, A.M., Wittkop, T., Sokolov, A., Graim, K., Funk, C., Verspoor, K., Ben-Hur, A., *et al.*: A large-scale evaluation of computational protein function prediction. Nature Methods **10**(3), 221–227 (2013)

[5] Yu, G., Domeniconi, C., Rangwala, H., Zhang, G.: Protein function prediction using dependence maximization. In: Proceedings of the 23rd European Conference on Machine Learning and Principles and Practice of Knowledge Discovery in Databases (ECML/PKDD), pp. 574–589 (2013)

[6] Lin, D.: An information-theoretic definition of similarity. In: Proceedings of the 15th International Conference on Machine Learning (ICML), pp. 296–304 (1998)

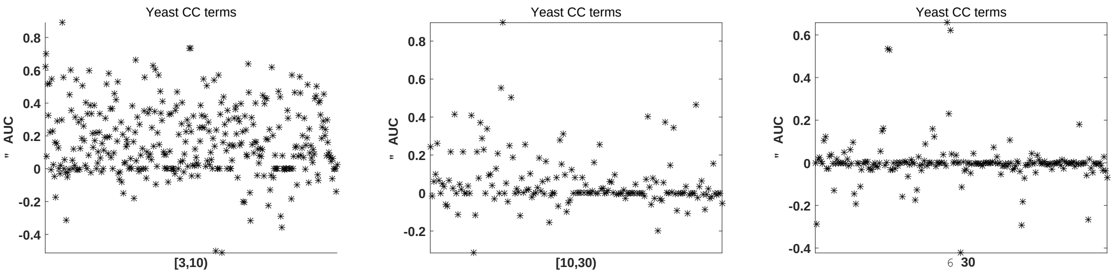

(a) [3,10) (b) [10,30) (c) ≥ 30

Figure S2: The AUC (Area Under the Curve) difference between dRW-kNN and ITSS on *Yeast* proteins annotated with *CC* terms in different sizes. [3,10) includes 359 terms, [10,30) includes 170 terms, and ≥ 30 includes 202 terms.

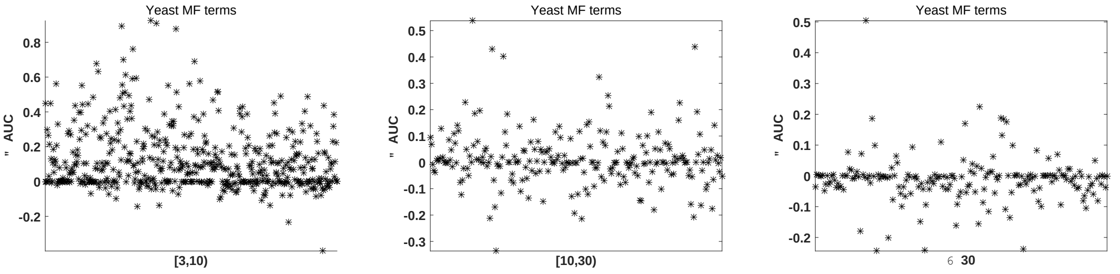

(a) [3,10) (b) [10,30) (c) ≥ 30

Figure S3: The AUC (Area Under the Curve) difference between dRW-kNN and ITSS on *Yeast* proteins annotated with *MF* terms in different sizes. [3,10) includes 546 terms, [10,30) includes 236 terms, and ≥ 30 includes 196 terms.

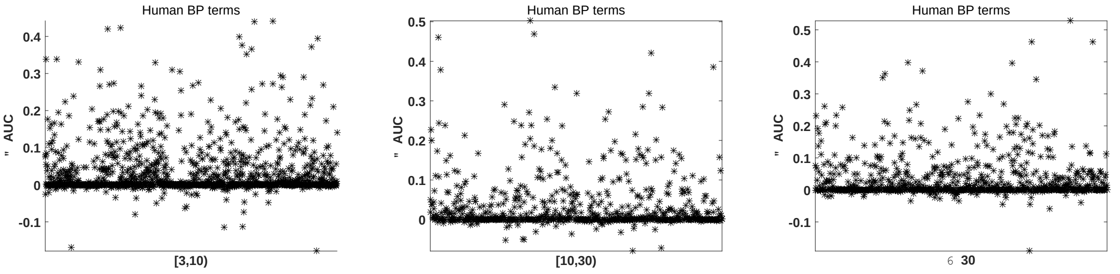

(a) [3,10) (b) [10,30) (c) ≥ 30

Figure S4: The AUC (Area Under the Curve) difference between dRW-kNN and ITSS on *Human* proteins annotated with *BP* terms in different sizes. [3,10) includes 3237 terms, [10,30) includes 1877 terms, and ≥ 30 includes 2180 terms.

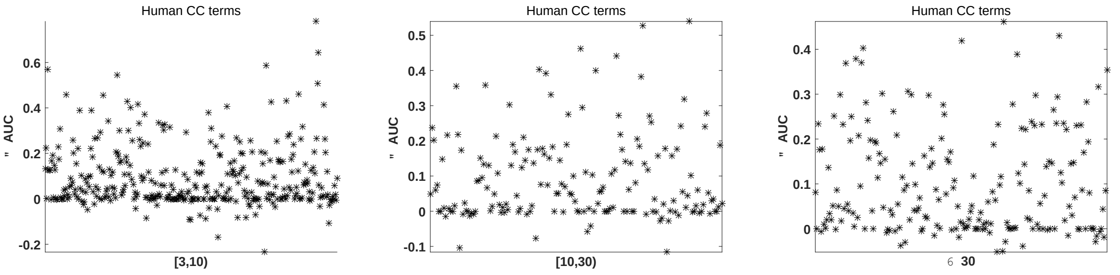

(a) [3,10) (b) [10,30) (c) ≥ 30

Figure S5: The AUC (Area Under the Curve) difference between dRW-kNN and ITSS on *Human* proteins annotated with *CC* terms in different sizes. [3,10) includes 414 terms, [10,30) includes 224 terms, and ≥ 30 includes 340 terms.

[7] Ferreira, J.o.D., Hastings, J., Couto, F.M.: Exploiting disjointness axioms to improve semantic similarity measures. Bioinformatics **29**(21), 2781–2787 (2013)

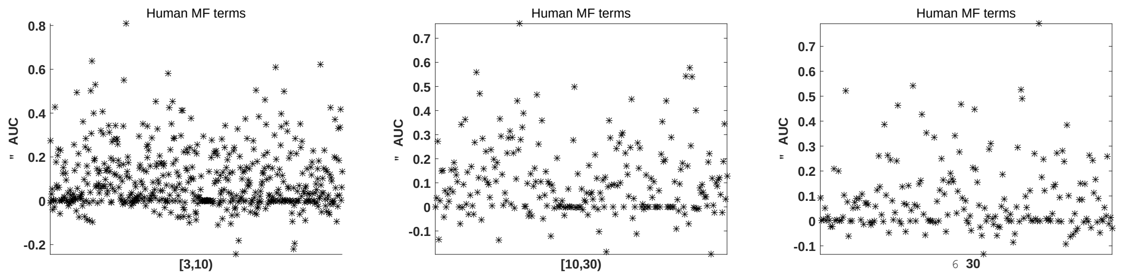

(a) [3,10) (b) [10,30) (c) ≥ 30

Figure S6: The AUC (Area Under the Curve) difference between dRW-kNN and ITSS on *Human* proteins annotated with *MF* terms in different sizes. [3,10) includes 943 terms, [10,30) includes 420 terms, and ≥ 30 includes 409 terms.

Table S6: Results of dRW, dRW-Corpus, dRW-Disjoint and dRW-E in predicting the missing *CC* functions of *Yeast* proteins, $|\mathcal{T}| = 731$ with $m = 3$. The numbers in **boldface** denote the best statistically significant performance (according to a t-test at 95% significance level).

Metric	dRW	dRW-Corpus	dRW-Disjoint	dRW-E
MacroF1	61.23±0.64	52.95±0.54	60.53±0.11	60.52±0.48
AvgROC	**96.31±0.07**	79.40±0.27	95.84±0.15	95.87±0.01
1-RankLoss	**97.64±0.03**	86.73±0.05	96.70±0.06	96.70±0.04
Fmax	**89.45±0.06**	88.49±0.04	88.74±0.03	88.67±0.01
RAccuracy	**38.21±0.51**	20.72±0.43	29.94±0.40	27.84±0.09
Coverage↓	**131.06±0.68**	518.26±2.41	170.54±1.96	169.60±0.17

Table S7: Results of dRW, dRW-Corpus, dRW-Disjoint and dRW-E in predicting the missing *MF* functions of *Yeast* proteins, $|\mathcal{T}| = 978$ with $m = 3$. The numbers in **boldface** denote the best statistically significant performance (according to a t-test at 95% significance level).

Metric	dRW	dRW-Corpus	dRW-Disjoint	dRW-E
MacroF1	55.71±0.13	44.80±0.39	55.63±0.29	55.23±0.80
AvgROC	**93.33±0.06**	77.99±0.32	**93.27±0.07**	93.08±0.02
1-RankLoss	**90.92±0.06**	80.31±0.17	90.49±0.12	90.07±0.06
Fmax	**73.70±0.03**	72.05±0.00	73.50±0.05	73.45±0.02
RAccuracy	**26.20±0.10**	11.59±0.34	19.67±0.16	19.77±0.27
Coverage↓	**204.25±1.66**	486.32±2.13	214.59±2.99	220.82±2.69

Table S8: Results of dRW, dRW-Corpus, dRW-Disjoint and dRW-E in predicting the missing *BP* functions of of *Human* proteins, $|\mathcal{T}| = 7294$ with $m = 3$. The numbers in **boldface** denote the best statistically significant performance (according to a t-test at 95% significance level).

Metric	dRW	dRW-Corpus	dRW-Disjoint	dRW-E
MacroF1	85.86±0.06	**86.53±0.05**	85.73±0.04	85.74±0.06
AvgROC	99.78±0.00	97.23±0.04	99.77±0.01	99.77±0.00
1-RankLoss	**99.21±0.01**	94.19±0.01	99.09±0.01	99.05±0.01
Fmax	94.07±0.01	94.02±0.00	94.07±0.01	94.07±0.01
RAccuracy	**28.47±0.21**	12.91±0.14	26.15±0.19	25.39±0.20
Coverage↓	**549.45±6.47**	4369.49±20.49	612.52±8.19	631.80±6.40

Table S9: Results of dRW, dRW-Corpus, dRW-Disjoint and dRW-E in predicting the missing *CC* functions of *Human* proteins, $|\mathcal{T}| = 978$ with $m = 3$. The numbers in **boldface** denote the best statistically significant performance (according to a t-test at 95% significance level).

Metric	dRW	dRW-Corpus	dRW-Disjoint	dRW-E
MacroF1	66.47±0.41	63.96±0.67	66.35±0.38	66.70±0.19
AvgROC	98.11±0.06	86.05±0.54	98.05±0.07	98.10±0.03
1-RankLoss	**96.36±0.02**	86.06±0.03	95.47±0.02	95.38±0.01
Fmax	**87.38±0.02**	86.96±0.02	87.31±0.01	87.23±0.01
RAccuracy	**31.46±0.11**	21.40±0.22	22.68±0.16	20.30±0.06
Coverage↓	**221.12±1.63**	728.64±0.86	273.32±0.73	276.86±1.05

Table S10: The results of dRW, dRW-Corpus, dRW-Disjoint and dRW-E in predicting the missing *MF* functions of *Human* proteins, $|\mathcal{T}| = 1772$ with $m = 3$. The numbers in **boldface** denote the best statistically significant performance (according to a t-test at 95% significance level).

Metric	dRW	dRW-Corpus	dRW-Disjoint	dRW-E
MacroF1	60.24±0.16	54.05±0.21	59.73±0.18	60.15±0.48
AvgROC	95.29±0.09	81.39±0.12	95.10±0.10	95.06±0.02
1-RankLoss	**91.93±0.04**	81.87±0.03	91.58±0.07	91.03±0.03
Fmax	**76.73±0.01**	75.15±0.01	76.60±0.02	76.51±0.04
RAccuracy	**28.29±0.11**	11.91±0.15	17.54±0.09	17.46±0.26
Coverage↓	**364.05±2.79**	922.08±3.99	382.98±2.64	404.94±0.42
